# Acceptance of the proposed social health insurance among government-owned company employees in Northwest Ethiopia: implications for starting social health insurance implementation

**DOI:** 10.1186/s13690-020-00488-x

**Published:** 2020-10-20

**Authors:** Abuneh Zemene, Adane Kebede, Asmamaw Atnafu, Tsegaye Gebremedhin

**Affiliations:** 1 University of Gondar Student Clinic, University of Gondar, Gondar, Ethiopia; 2 Department of Health Systems and Policy, Institute of Public Health, College of Medicine and Health Sciences, University of Gondar, P. O. Box: 196, Gondar, Ethiopia

**Keywords:** Social health insurance, Acceptance, Government-owned company, Ethiopia

## Abstract

**Background:**

Ethiopia is currently planning to introduce Social Health Insurance (SHI) that will lead to universal health coverage and assist a country to achieve its health system’s objectives and to prevent the catastrophic health expenditure. But there is no evidence until now about the level of acceptance of the proposed SHI among government-owned companies’ employees. Therefore, this study was intended to assess the acceptance of SHI and associated factors among government-owned companies’ employees in northwest Ethiopia.

**Methods:**

An institution-based cross-sectional study was conducted from February 1 to April 30, 2019. A randomly selected 541 government-owned companies’ employees were participated in the study. A pretested self-administered structured questionnaire was used that consisted sociodemographic and economic, health status-related factors, attitude (measured by 12 items), organizational related factors and knowledge about SHI (measured by 11 items). Finally, binary logistic regression analysis was performed and in the multivariable logistic regression analysis, a significant level at a *p*-value of < 0.05 and Adjusted Odds Ratio (AOR) with 95% confidence interval (CI) were used to identify factors statistically associated with SHI acceptance.

**Results:**

Overall, 32% (95% CI: 27.7–36.2) of the government-owned companies’ employees accepted the proposed Ethiopian SHI scheme. Self-perceived health status (AOR: 8.55, 95% CI: 2.69–27.13), heard about SHI (AOR: 1.69, 95% CI: 1.12–2.54), coverage of medical healthcare cost (AOR: 0.60, 95% CI: 0.39–0.92), work experience (AOR: 0.49, 95% CI: 0.26–0.89) and quality of healthcare service at the facilities (AOR: 0.17, 95% CI: 0.04–0.71) were significantly associated with acceptance of SHI among government-owned companies’ employees.

**Conclusions:**

One-third of the study participants accepted the proposed Ethiopian SHI scheme. Self-perceived health status, quality of healthcare service at health facilities, coverage of the medical cost by their organization, heard about SHI and work experience were the factors that affect acceptance of the proposed SHI among the government-owned company employees. Therefore, policymakers should devise a plan to promote the benefit packages of SHI for the formal sector employees to start the implementation.

## Background

Universal health coverage (UHC) has been championed by health policymakers around the world. However, reality lags far behind ensuring UHC in low-and middle-income countries (LMICs) [1] due to a range of factors [2–6]. The Social Health Insurance (SHI) initiatives have originated from Germany swept across Asia, Latin America and Africa to contribute for the reduction of healthcare finance scarcity and improvements of poor health. In 2005, the World Health Assembly designed SHI as a strategy for mobilizing more resources for health, pooling risk, providing equitable access to healthcare for the poor and delivering better quality healthcare [7, 8].

Social health insurance is a form of organizational mechanisms for raising and pooling funds to finance and manage health services that will lead to UHC and assist a country achieve its health system’s objectives [9]. Typically, the model, working people and their employers, as well as the self-employed, pay contributions that cover a package of services available to the insurees and their dependents. Most of the low- and middle-income countries face difficulties in funding healthcare. While those nations declare similar goals to provide their citizens with access to a reasonable quality of healthcare and to prevent health-causing impoverishment towards the achievement of UHC, the reality is starkly different and health status is still not improved [7].

Ethiopia endorsed a healthcare financing strategy since 1998 that envisioned a wide range of reform initiatives to form the recent health system structure. Since the Ethiopian healthcare system is under tremendous reform [10], the health insurance (HI) strategy designed to promote equitable access to sustainable quality healthcare, increase financial protection and enhance social inclusion for the majority of Ethiopian families in the health sector [11]. Health insurance was initially introduced in the form of community-based since 2010, whereas the SHI is not yet introduced [12, 13]. The community-based health insurance (CBHI) schemes designed to provide financial protection against the costs of health care and expand access to modern health-care services to the informal and rural sectors, whereas SHI is for the formal sectors. The HI benefit packages includes all family health services and curative healthcare services package at all levels. The curative care covers outpatient and inpatient services including treatment of acute illness and injuries at all levels. Whereas non-life-threatening treatment such as cosmetic surgery, ornamental dental replacement etc., were not included [14]. Even though SHI the first kind of scheme which has been designed for those who are engaged in the formal sectors, it is not yet started.

Despite the significant health improvements in the last fifteen years, the African population still has poor health status [15]. In many sub-Saharan African countries, studies show that the poor bear the highest burden of diseases and high levels of catastrophic health expenditures and over 90% of health-related shocks occur in LMICs [16, 17]. The most significant constraints against rapid scale-up of healthcare are prevailing inadequacy and inefficiency in resource mobilization and allocation for health [15, 18]. The Ethiopian society is subject to increasing user fees for healthcare services because no formal pre-paid financial mechanism like SHI that allows cross-subsidization of the poor by the rich and the sick by the healthy to risk pooling and sharing among different willing individuals before and still know [7]. In Ethiopia, out-of-pocket (OOP) expenditures accounted for 33% of total health spending. Moreover, risk protection systems during an illness are low and overall health status are very poor [19, 20].

In Ethiopia, there has been a growing interest in improving healthcare financing through the insurance system and different measures have been undertaking to implement SHI in the formal sector, though not started and also the factors are not identified, particularly for these government-owned companies [21].

Worldwide, a range of studies identified factors that affect the willingness to pay for SHI among civil servants. Studies revealed from Vietnam and Namibia primary level education and beyond has positively impacted the probability of respondents to accept SHI and also had significant value and directly related to the willingness to pay (WTP) SHI [22, 23]. A study in Bangladesh indicated that, in the formal sectors, educated household heads are more willing to pay for health insurance than non-educated [24]. Findings in Iran showed that the WTP for HI was positively correlated with income, education, employment status of household heads and the number of insured family members [25]. A study in Debub Bench district, southwest Ethiopia shows that family size was one of the determinant factors for HI uptake [26].

Moreover, a study in Sarawak Malaysia revealed that occupation, level of education, family size, marital status, family income and treatment preference were the potential predictors for willingness to accept HI by 48.1% [27]. A study conducted in that of china revealed that health status is a determinant factor in people’s decisions to enroll in HI. People with low health status are more willing to accept HI schemes than healthier ones [28]. The study conducted in Portugal showed that respondents’ health-related behaviors play an essential role in their willingness to subsidize social well-being through healthcare expenditures [29]. The research conducted in central Vietnam Chronic disease was related to WTP only at a copayment level of 20% [30]. Identifying the voluntariness of beneficiaries is an important issue to start a HI program [31].

Assessing the acceptance of SHI and associated factors can help the government to implement the SHI in the government-owned companies, which will be essential to pool the risk of out of payment among formal sector employees. Therefore, this study aimed to assess the magnitude of acceptance of the SHI schemes and associated factors among government-owned company employees in northwest Ethiopia.

## Methods

### Study design and settings

An institution-based cross-sectional study was conducted in Gondar city, northwest Ethiopia from February 1 to April 30, 2019. Gondar city is located 733 km to the north of Addis Ababa, the capital of Ethiopia and nearly 360,600 people resides [32]. The government-owned companies in Gondar city are the commercial banks of Ethiopia, Ethio-Telecom, Dashen brewery factory, Ethiopian electric power, Amhara saving and credit institution, Ethiopian airline, Gondar *bikil* factory, and Ambasel trade organization [33, 34]. There are a total of 1472 employees in those companies. All employees in government-owned companies’ in Gondar city were the source population, whereas employees who were working in government-owned companies during the data collection period were the study population. Those employees working for at least 3 months in their respective companies were included, whiles those foreign in citizenship were excluded from the study.

### Sample size and sampling procedures

The sample was calculated for the acceptance and factors to take the largest sample size. The sample size for acceptance was calculated using the single population proportion formula (n $=\frac{P\left(1-P\right){\left({Z}_{\alpha /2}\right)}^2}{d^2}$) and assuming a 69.8% proportion (P) of acceptance of social health insurance taken from another similar study [35], 4% margin of error (d), 95% confidence level (CI) and 10% non-response rate that yielded a sample of 557.

The sample size for the factors was determined using the power approach by considering 80% power, 95% CI, 10% non-response rate, and significant variables from studies conducted in those of Wolayta Sodo [36] and Debre-Markos [35]. Those significant variables were marital status (63.7%), educational status (81%), ever heard about SHI (83%) and family size (74.8%), and the calculated sample size was 108, 117, 161 and 271, respectively. We took the largest sample size, which was 557.

The sample was proportionally allocated to their number of employees after reviewing the lists from each government-owned company. The final study participants were selected using the simple random sampling technique (lottery method) from their list and self-administered interview was conducted.

### Variables and measurements

The dependent variable of the study was acceptance of SHI. The Ethiopian government proposed the SHI which is not yet started, in which every government employee will pay 3 % of his/her monthly salary. Based on the proposed SHI, the participant was asked whether they accept or not accept it [35].

A Government-owned companies (state-owned companies/non-profit organizations) in Ethiopia are the legal entity that undertakes commercial activities on behalf of an owner government. Moreover, they are non-profit organizations with distinct legal forms and that they are established to operate in commercial affairs and they have public policy objectives.

Socio-demographic and economic factors**,** health status-related factors, attitude, organizational related factors, and knowledge about SHI were the independent variables of the study. The sociodemographic and economic factors were sex (male, female), age (20–24, 25–29, 30–34, 35–39, 40+), ethnicity (Amhara, Tigre, Oromo, others), religion (Orthodox, Muslim, Protestant), marital status (single, married), educational status (diploma, degree, others) and family size (1–3, 4+), monthly salary (≤7430, 7431–9490, 9491–12,700, > 12,700) and work experience (≤5, 6–8, 9–12, 13+). Educational status was categorized based on the Ethiopian education level; Diploma means graduated from a college after 2 years of study, degree includes both Bachelor and Master degree and certificate means a year or less completed training on a specific profession and or field.

Health status related factors includes the presence and or history of chronic illness and perceived health status (poor, good, very good).

Organization related factors were the types of business (company), covering the cost of health care (yes, no) and position (business officer, accountant, technician, secretary, manager).

Knowledge of social health insurance was assessed by using a set of 11 questions about social health insurance and the right answer was given a value of 1 and 0 for those incorrect answers. Respondents who had a score greater than or equal to the mean score (mean score: 5.1) were considered as having “good knowledge” about SHI, otherwise as “poor knowledge” [35, 37]. The Cronbach alpha value for the eleven items of questions was 0.84.

Attitude towards SHI was measured using 12 items of questions with a five-point Likert scale (1: strongly disagree, 2: disagree, 3: neutral, 4: agree, and 5: strongly agree). The sum of these items yielding a total score of minimum 12 and maximum 60 with a mean score of 37.0 and also the Cronbach’s alpha score was 0.78. Accordingly, if participants scored ≥37.0, we considered them as having “good attitude”, otherwise as “poor attitude” about SHI [38].

### Data collection tools and procedures

A structured self-administered questionnaire was prepared by reviewing related studies [38], which consists of socio-demographic characteristics, economic status, health status, knowledge, attitude, and acceptance of SHI. The questionnaires were first prepared in English and then translated into the local language (Amharic) and finally back to English to ensure consistency.

Six trained certificates in diploma nurses and two Bachelor’s degree graduate nurses were recruited from the University of Gondar specialized hospital as data collectors and supervisors, respectively. A pretest was conducted among 70 participants in the government-owned company in Bahir Dar city (the nearby city). Then necessary amendments from the pretest findings were incorporated on the tool before the actual data collection. The data collectors were supervised daily, and the consistency and completeness of data were checked by the principal investigator every night.

### Data processing and analysis

The completed data were entered into Epi-Info version 7 and exported to Statistical Package for Social Sciences (SPSS) version 23 for analysis. Descriptive statistics such as frequency, mean, median and standard deviation were used to show a clear picture of the respondents. Bivariable and multivariable logistic regression analyses were performed. In the bivariable logistic regression analysis, variables having a *p*-value of less than 0.2 were candidate for multivariable logistic regression analysis. The significance of association in the final model was determined using a *p*-value of < 0.05 and adjusted odds ratio (AOR) with 95% confidence level (CI) after model fitness was checked using the Hosmer–Lemeshow goodness-of-fit test (*p* = 0.25).

## Results

### Sociodemographic and economic characteristics of participants

A total of 541 government-owned company employees were participated in the study, with a response rate of 97.3%. Of the total respondents, 60.8% were males; the majority (88.5%) were Amhara. Religious preference for 83.4 and 6.5% of the participants were Orthodox christian and Protestant, respectively; 67.7% were married; 78.9% were degree holders, while 21.1% were diploma holders and lower. The mean age of the study participants was 33.7 (SD ± 6.2) years and the mean family size was 3 (SD ±1.6). The median salary was 9490 (IQR: 2000–28,000) Ethiopian Birr (ETB) ($1 USD = 29.1 ETB) (Table 1).

**Table 1:** Socio-demographic and economic characteristics of participants in northwest Ethiopia, May 2019 (*n* = 541)

Variables	Category	Frequency (n)	Percentage (%)
Sex	Male	329	60.8
	Female	212	39.2
Age in years	20–24	9	1.6
	25–29	160	29.6
	30–34	156	28.8
	35–39	102	18.9
	≥40	114	21.1
Ethnicity	Amhara	486	89.8
	Tigre	21	3.9
	Oromo	27	5.0
	Others	7	1.3
Religion	Orthodox	451	83.4
	Muslim	55	10.1
	Protestant	35	6.5
Marital status	Married	366	67.7
	Single	175	32.3
Family size	1–3	350	64.7
	≥4	191	35.3
Positions	Business officers	341	63.0
	Accountant	96	17.8
	Technicians	58	10.7
	Secretariats	38	7.0
	Managers	8	1.5
Level of education	Diploma	59	10.9
	Degree	427	78.9
	Others^a^	55	10.2
Work experiences in years	≤ 5	169	31.2
	6–8	112	20.7
	9–12	137	25.3
	≥ 13	123	22.8
Monthly salary (ETB)	≤ 7430	136	25.0
	7431–9490	137	25.4
	9491–12,700	133	24.6
	> 12,700	135	25.0

^a^Others: Certificates and non-certificates, ETB: Ethiopian Birr

### Health and health-related characteristics of study participants

Participants perceived their health status as very good (22.6%), good (71.5%), or poor (5.9%). Nearly 37% of the participants responded that the health services provided at the health facilities have good quality. Five percent of the study participants had a family history of chronic medical illness (diabetes mellitus, hypertension, cardiac and others). Of the total study participants, 9.6% of them had a history of at least one episode of illness in the last 6 months. Of these who had a history of illness, all of them were received treatment and 48.1 and 51.9% were from governmental and private health institutions, respectively. Regarding medical bills of treatment (for those who had a history of illness within 6 months of the survey), 51.9, 36.5 and 11.5% of the medical expense was covered from out of pocket, freely and borrowed, respectively.

### Knowledge and attitudes of participants about social health insurance

The mean score of participants’ knowledge about SHI was 5.1 (SD ± 1.77). Nearly 41% of the respondents had good knowledge; 49.9% respondents ever heard about SHI. Of those, 75, 31.9, 17.0 and 9.3% were from television, insurance agents, conferences and meetings and others, respectively. Only 146 (27%) participants had another type of health insurance previously.

The mean score of participants’ attitude about SHI was 37.0 (SD: ± 6) and less than half (47.7%) of the participants had good attitude about SHI. Moreover, for the implementation scheme/decision of SHI by the Ethiopian government, about 298 (55.1%) were agreed to appertain, 88 (16.2%) were neutral and 155 (28.7%) disagreed.

### Acceptance of the proposed social health insurance

Overall, 32% (95% CI: 28–36) of the government-owned companies’ employees were accepting the proposed SHI. The acceptance of the proposed SHI among bank and Amhara saving and credit institution employees were 10 and 4.4%, respectively (Table 2).

**Table 2:** Acceptance of the social health insurance status among government-owned companies’ employees in northwest Ethiopia, May 2019 (*n* = 541)

Business type	Frequency (n)	Percentage (%)
Bank	54	10.0
Airline, Dashen brewery and Ambasel	35	6.5
Ethio-electric	34	6.3
Ethio-tele	26	4.8
Amhara saving and credit institution	24	4.4
Total	173	32.0

Of those who did not accept the proposed SHI, 17% of them were due to lack of trust to get all types of treatment by the insurance, free treatment gained from their organization and lack of confidence from the implementation of the program. The other reasons were unable to pay the monthly premium, poor quality of healthcare services, government/organizational responsibility, feeling being health and ability to pay out of pocket payment for health costs in 18.5, 13.5, 12.8, 11.8, 9.8 and 0.9%, respectively.

### Factors associated with the acceptance social health insurance

In the multivariable logistic regression analysis, self-perceived health status, quality of healthcare service, type of employer organization that covered medical healthcare cost, heard about SHI, and work experience were significant variables with acceptance of the proposed SHI among government-owned companies’ employees.

Accordingly, those who perceived their health status as poor and good were 8.56 (AOR = 8.56, 95% CI: 2.70–27.13) and 1.86 (AOR = 1.86, 95% CI: 1.12–3.10) times more likely to accept the proposed SHI than those who perceived very good. Those study participants who ever heard about SHI were 1.69 times more likely to accept the SHI than those who did not hear (AOR = 1.69, 95% CI: 1.12–2.55). Those participants’ medical healthcare costs covered by their organization were 40% less likely to accept the SHI compared to their counterparts (AOR = 0.60, 95% CI: 0.39–0.92). Those who had work experience greater than or equal to 13 years were 51.0% less likely to accept the proposed SHI compared to those who had less than or equal to 5 years experiences (AOR = 0.49, 95% CI: 0.27–0.89). Moreover, those who perceived that the quality of services provided at the health facilities was poor were 82.6% less likely to accept the proposed SHI compared to those who perceived good quality of healthcare service (AOR = 0.17, 95% CI: 0.04–0.72) (Table 3).

**Table 3:** Bivariable and multivariable logistic regression analysis for acceptance of social health insurance among government-owned companies’ employees in northwest Ethiopia, May 2019 (*n* = 541)

Variables	Response	Acceptance of SHI		COR (95% CI)	AOR (95% CI)
		No n (%)	Yes n (%)		
Marital status	Married	241 (65.8)	125 (34.2)	1	1
	Single	127 (72.6)	48 (27.4)	0.73 (0.49–1.08) *	0.63 (0.39–1.01)
Work experience in years	≤5	115 (68.1)	54 (31.9)	1	1
	6–8	65 (58.0)	47 (42.0)	1.54 (0.94–0.25) *	1.38 (0.80–2.38)
	9–12	95 (69.3)	42 (30.7)	0.94 (0.58–1.53)	0.71 (0.41–1.24)
	≥13	93 (75.6)	30 (24.4)	0.69 (0.41–1.16) *	0.49 (0.27–0.89) **
Income in ETB	≤7430	90 (66.2)	46 (33.8)	1	1
	7431–9490	94 (68.6)	43 (31.4)	0.89 (0.54–1.49)	0.88 (0.51–1.54)
	9491–12,970	85 (63.9)	48 (36.1)	1.11 (0.67–1.82)	1.28 (0.67,2.41)
	> 12,970	99 (73.3)	36 (26.7)	0.71 (0.42–1.20) *	0.82 (0.41–1.66)
Self-rate perceived health status	Very Good	92 (75.4)	30 (24.6)	1	1
	Good	261 (67.4)	126 (32.6)	1.48 (0.93–2.35) *	1.86 (1.12–3.10) **
	Poor	5 (29.4)	12 (70.6)	7.36 (2.40–22.59) *	8.55 (2.69–27.13) **
	Bad	10 (66.7)	5 (33.3)	1.53 (0.49–4.84)	1.22 (0.35–2.21)
Perceived quality of health service	Poor	240 (70.0)	103 (30.0)	0.29 (0.08–1.04) *	0.17 (0.04–0.72) **
	Good	128 (64.6)	70 (35.4)	1	1
Heard about SHI	Yes	194 (71.6)	77 (28.4)	1.39 (0.97–1.99) *	1.69 (1.12–2.55) **
	No	174 (64.4)	96 (35.6)	1	1
Organization covers healthcare cost	Yes	125 (51.9)	116 (48.1)	0.43 (0.53–1.13) *	0.60 (0.39–0.92) **
	No	143 (71.5)	57 (28.5)	1	1
Knowledge towards SHI	Not knowledgeable	228 (71.3)	92 (28.7)	1	1
	Knowledgeable	140 (63.3)	81 (36.7)	1.43 (0.99–2.07) *	1.20 (0.78–1.84)
Attitude towards SHI	Good	168 (65.1)	90 (34.9)	0.78 (0.54–1.11) *	0.74 (0.50–1.10)
	Poor	200 (70.7)	83 (29.3)	1	1
Goodness-of-fit test (X^2^ = 70.28, *p*-value = < 0.001)					

*AOR* Adjusted odds ratio, *COR* Crude odds ratio, *ETB* Ethiopian Birr, *Statistically significant at *p*-value < 0.2, **statistically significant at *p*-value < 0.05, 1: reference category, *SHI* Social Health Insurance

## Discussion

The study showed that 32% of the respondents were accepting the proposed SHI scheme. The result was lower than these of studies conducted at Mekelle city (85.3%) [39], Wolaita Sodo (71.1%) [36], and at Debre-Markos (68.9%) [35] in which participants were willing to join the proposed SHI. This difference might be due to the organizational behavior which covered medical healthcare cost and might be due to study type. But higher than that of a study done at St. Paul’s Hospital Millennium Medical College Addis Ababa Ethiopia among health workers in which 17% were willing to pay for SHI [38] and Bahirdar city, northwest Ethiopia among civil servants in which 33.2% were willing to pay 1% of their monthly income and only 17.3% of participants were willing to pay ≥3% of their monthly salary [40]. The difference might be due to the study period, trust in the HI agency and perceived quality of healthcare service.

Even if this result was smaller compared to other studies, it was encouraged to start the proposed SHI scheme because those who accept the proposed SHI (3% of monthly salary) are nearly one-third which is good to start a pilot implementation as proposed by the Ethiopian government [31, 41]. However, it was not matched what the government needs according to the newly proposed SHI scheme which needs more effort to resolve the factors affecting acceptance and promote the benefit of the insurance. Indeed, the acceptance level of SHI in other sectors like that of healthcare is higher which indicates a growing interest of participation [42].

In our study, self-perceived health status, quality of healthcare service, type of employer organization that covered medical healthcare cost including family members, having information about SHI and work experience were statistically associated variables with the acceptance of SH. These variables could be a possible result of the majority of the study participants not accepting to pay for the proposed SHI scheme.

The finding revealed that those who perceived their health status was poor and good were 8.5 and 1.86 times more likely to accept the proposed SHI than those who perceived that their health status was very good, respectively. This result was comparable with that of a study findings in Portugal in which health-related factor was one of the causes to determine the acceptance of healthcare expenditure [29]. The possible explanation might be seeking treatment during illness to reduce the economic burden of healthcare show signs of being risk-averse. However, different from a study done from Vietnam, a negative relationship was seen between chronic disease and WTP for SHI in the scenario of a 20% copayment level [30]. Moreover, it is inconsistently in Iran in which health status were not having significant value [25]. This difference might be due to fear of copayment level or might be by the economic burden of healthcare treatment cost or might be the user’s fee payment level difference in different nations and income intensity difference. The other possible explanation might be those who have poor health status might have frequent facility visits for their medical treatment and incur more cost that prone willing to pay for SHI to be protected from incurring more cost.

In this study, participants who ever heard about SHI were 1.69 times more likely to accept SHI than those who never heard about SHI. Our result was in line with these of study’s findings in Ebonyi State, Nigeria 30.3% of the respondents agreed to have the scheme were heard about national health insurance (NHI), and in Osun State Nigeria, 28.9% of participants WTP were heard and aware of the NHI [37, 43]. Furthermore, it is related to a study done in Vietnam; almost all respondents (91.8%) WTP for SHI were heard about SHI scheme [30] and in Wolayita Sodo, Ethiopia individuals who heard about HI scheme were 2.5 times more likely to willing to pay compared to those who have never heard of HI scheme [36]. Similarly, in Bahir Dar city, Ethiopia participants who have information on SHI were 2.45 more likely willing to pay than those who did not have information [40]. This difference might be a promotion difference from the HI agency. These results demonstrate that participants who ever heard about SHI were more likely to WTP for SHI and having the value of the HI scheme.

The study revealed that participants who perceived that the quality of healthcare service was good were 82.6% less likely to accept the SHI compared to those who perceived that the quality of healthcare service was poor. The result is in line with that of a study done in Mekelle City in which the quality of health service was an essential issue in the focusing group and for dissatisfaction [39]. A study conducted at St. Paul’s Hospital Millennium Medical College, Addis Ababa, revealed that those health workers who believed that the SHI scheme improves the quality of services provided by healthcare givers were 2.7 times more likely to willing to pay than those who did not believe in the scheme [38]. This result is also related to the study done in Malaysia treatment preference, as was one of the significant variables for acceptance of SHI [27]. The possible reason might be due to treatment preference, expect good quality of healthcare service under the SHI scheme, and inability to pay medical bills. This inform us that there was a significant association between willingness to pay and perceived the quality of healthcare service under the SHI scheme and quality of health services in public health facilities. It also indicates that participants who had poor quality of healthcare service would expect good quality of healthcare service by SHI scheme, and participants who had a good quality of healthcare service would prefer to be treated in public health facilities. So, positively associated with acceptance of the SHI and on quality of healthcare service might be seen in this study.

Our finding demonstrated that participants who covered medical healthcare expenditure by employer organization were 40% less likely to accept SHI compared to those who had not covered medical healthcare cost by employer organization. Moreover, 36.9% of the medical bills were covered by the organization. This result was related to research done in Sierra Leone, in which 61% of respondents in treatment choice were in public hospitals and Sarawak Malaysia, 14% of participants believed that the government’s responsibility to bear treatment cost. The same reason in Osun state Nigeria, 12.4% were non-willingness to pay and believed that the government alone should pay for healthcare service expenses and at St. Paul’s Hospital Millennium Medical College of Addis Ababa, 13% of the respondents stated that healthcare services should be the responsibility of the government [27, 37, 38, 44, 45]. The possible reasons might be due to Government’s responsibility to bear the treatment cost; the possible coverage of medical healthcare costs made them consider insured and risk-neutral (misconception for SHI) employees might not feel a financial burden for seeking treatment.

In this study those who had work experience greater than or equal to thirteen years were decreased by 50.9% compared to those who had work experience less than or equal to 5 years. This result was related with studies done in Malaysia on academic staff, senior staffs were 18.7% less likely to accept SHI than junior staffs [44], but this result was inconsistent to studies done at St. Paul’s Hospital Millennium Medical College, Addis Ababa on which work experience was no associated factors [38]. The possible reason might be due to study participants who had other work experiences that might not feel a financial burden for seeking treatment owing to get hold of a better salary and might be due to a premium level. Additionally, those with more work experience may have more income and have the ability to pay for private health facility services other than visiting public health facilities.

Among those government-owned companies’ employees accepting the proposed SHI majority (31.2%) were from bank. The possible reason might be a difference in medical bill cost coverage by employer organizations. The study was comprised mostly male respondents (57.8%) to accept SHI and the result was similar to studies done at St. Paul’s Hospital Millennium Medical College, Addis Ababa, that 54% of study participants were males [38] and in Nigeria 58% participants were male [43]. However, our multivariable logistic regression analysis did not show religion, respondents’ monthly salary, income, age, family size, marital status and educational status as a predictor variable. Contrary to this, a study from Malaysia, Iran, South Sudan, and Nigeria [18, 25, 37, 44] in which such factors influenced individual decision to pay for the social health insurance scheme which this could be due to economic difference or might be the cultural status difference.

### Limitation of the study

The study used a self-administered questionnaire which was difficult to probing for additional information which can subsequently increases skipping and not responded to all the questions. To minimize this, we have used valid and with clear items of questionnaires prepared in local language that every respondent can understand easily. In this study, the discussion was made majorly with the willingness to pay and join social health insurance, because to the best knowledge of the researchers, there is a limited study about the acceptance of the proposed SHI scheme among the formal sectors. It is known that acceptance of the insurance comes before the willingness to join and pay, so we tried to discuss the incongruency in detail. The other possible limitation of the study was it does not use qualitative, which needs exploring the reason why they did not accept the proposed SHI scheme among the government-owned company employees compared to other formal sector employees.

## Conclusions and implications

Only one-third of the study participants accepted the proposed Ethiopian SHI scheme. Perceived health status and quality of healthcare service, covering of medical cost, prior information about SHI and work experience has significantly affected the acceptance of the proposed SHI among the government-owned company employees. Therefore, the Ethiopian HI agency should engage the potential stakeholders to increase the benefit of the insurance strategies among the formal sectors, particularly in the government-owned company employees as they are in the higher salary scale of our country; they will contribute more to pool the risk through their participation.

Even though SHI for government-owned company employees in the formal sectors is not yet implemented in Ethiopia, it is better to know the employees’ acceptance and the factors which inhibit the implementation. So, researchers should conduct mixed-method researches to address various inquiries on the non-acceptance of social health insurance among formal sector employees and government-owned company employees, which will help to start the implementation.

## Data Availability

Data will be available upon reasonable request from the corresponding author.

## Abbreviations

AOR: Adjusted Odds Ratio
CI: Confidence Interval
COR: Crude Odds Ratio
LMICs: Low-and-Middle Income Countries
SHI: Social Health Insurance
SPSS: Statistical Packages for Social Sciences
UHC: Universal Health Coverage
WHO: World Health Organization
WTJ: Willingness to Join
WTP: Willingness to Pay

## References

[1] Wong J (2015). Achieving universal health coverage. Bull World Health Organ.

[2] Kruk ME, Gage AD, Joseph NT, Danaei G, García-Saisó S, Salomon JA (2018). Mortality due to low-quality health systems in the universal health coverage era: a systematic analysis of amenable deaths in 137 countries. Lancet.

[3] Ranabhat CL, Jakovljevic M, Dhimal M, Kim CB. Structural factors responsible for universal health coverage in low-and middle-income countries: results from 118 countries. Front Pub Health. 2019;7:414. 10.3389/fpubh.2019.00414 PMC6985282 32039128

[4] Watkins DA, Jamison DT, Mills A, Atun R, Danforth K, Glassman A, Horton S, Jha P, Kruk ME, Norheim OF (2017). Universal health coverage and essential packages of care.

[5] Moreno-Serra R, Smith PC (2012). Does progress towards universal health coverage improve population health?. Lancet.

[6] Reich MR, Harris J, Ikegami N, Maeda A, Cashin C, Araujo EC, Takemi K, Evans TG (2016). Moving towards universal health coverage: lessons from 11 country studies. Lancet.

[7] Hsiao W, Shaw RP (2007). Social health insurance for developing nations: the World Bank.

[8] World Health Report (2010) Priyanka Saksena KX, Varatharajan Durairaj (2010). The drivers of catastrophic expenditure.

[9] Doetinchem O, Carrin G, Evans D (2010). Thinking of introducing social health insurance? Ten questions. World health report.

[10] Zelelew H (2014). Health care financing reform in Ethiopia: improving quality and equity. Bethesda (MD).

[11] Feleke S, Mitiku W, Zelelew H, Ashagari T (2015). Ethiopia’s community-based health insurance: a step on the road to universal health coverage.

[12] Mebratie AD, Sparrow R, Yilma Z, Alemu G, Bedi AS (2015). Enrollment in Ethiopia’s community-based health insurance scheme. World Dev.

[13] Mebratie AD, Sparrow R, Yilma Z, Abebaw D, Alemu G, Bedi A (2013). Impact of Ethiopian pilot community-based health insurance scheme on health-care utilisation: a household panel data analysis. Lancet.

[14] Birara D (2018). Reflections on the health insurance strategy of Ethiopia.

[15] Health FDRoEMo: Health Sector Development Program IV, 2010/11—2014/15 (2010). Ministry of Health Addis Ababa.

[16] Fenny AP, Yates R, Thompson R (2018). Social health insurance schemes in Africa leave out the poor. Int Health.

[17] Xu K, Evans DB, Carrin G, Aguilar-Rivera AM, Musgrove P, Evans T (2007). Protecting households from catastrophic health spending. Health Aff.

[18] Basaza R, Alier PK, Kirabira P, Ogubi D, Lako RLL (2017). Willingness to pay for National Health Insurance Fund among public servants in Juba City, South Sudan: a contingent evaluation. Int J Equity Health.

[19] Ali EE (2014). Health care financing in Ethiopia: implications on access to essential medicines. Value in Health Reg Issu.

[20] Federal Democratic Republic of Ethiopia Ministry of Health (2017). The Ethiopia Sixth Health Accounts, 2013/14.

[21] Atafu A, Kwon S (2018). Adverse selection and supply-side factors in the enrollment in community-based health insurance in Northwest Ethiopia: a mixed methodology. Int J Health Plann Manag.

[22] Lofgren C, Thanh NX, Chuc NT, Emmelin A, Lindholm L (2008). People's willingness to pay for health insurance in rural Vietnam. Cost Effectiveness Resour Allocation.

[23] Emily G, Jacques V: Willingness to Pay for Health Insurance: An Analysis of the Potential Market for New Low Cost Health Insurance Products in Namibia. Center for Disease Control and Prevention\National Institute for Occupational Health & Safety (USA).

[24] Ahmed S, Hoque ME, Sarker AR, Sultana M, Islam Z, Gazi R, Khan JA (2016). Willingness-to-pay for community-based health insurance among informal Workers in Urban Bangladesh. PLoS One.

[25] Nosratnejad S, Rashidian A, Mehrara M, Sari AA, Mahdavi G, Moeini M (2014). Willingness to pay for social health Insurance in Iran. Global J Health Sci.

[26] Haile M, Ololo S, Megersa B (2014). Willingness to join community-based health insurance among rural households of Debub Bench District, bench Maji zone, Southwest Ethiopia. BMC Public Health.

[27] Azhar A, Rahman MM, Arif MT (2018). Willingness to pay for health Insurance in Sarawak, Malaysia: a contingent valuation method. Bangladesh J Med Sci.

[28] Bärnighausen T, Liu Y, Zhang X, Sauerborn R (2007). Willingness to pay for social health insurance among informal sector workers in Wuhan, China: a contingent valuation study. BMC Health Serv Res.

[29] Borges A, Reis A, Anjos J (2017). Willingness to pay for other individuals' healthcare expenditures. Public Health.

[30] Nguyen LH, Hoang ATD (2017). Willingness to pay for social health insurance in Central Vietnam. Front Public Health.

[31] Ethiopia Dro: Ministory of ECO social health insurance regulation number 2011/2012 Federal Negarit gazeta 2011/2012.

[32] EthioVisit.com (2019). Ethiopia Administrative Regions, Cities and Population.

[33] Government-owned companies in the Ethiopia (2017). Category: Government-owned companies of Ethiopia.

[34] Ngubane TB (2018). An analysis of the representation of female and male politicians during the 2016 South African local government elections: a case study of the Pietermaritzburg daily newspaper, The Witness.

[35] Abebaw B, Jara D, Asmamaw T, Chanie T. Willingness to Pay for the Newly Proposed Social Health Insurance Scheme and Associated Factors Among Civil Servants in Debre Markos Town, North West Ethiopia, 2015. Med Res Clin Case Rep. 2018;2(2):164–77.

[36] Agago TA, Woldie M, Ololo S (2014). Willingness to join and pay for the newly proposed social health insurance among teachers in Wolaita Sodo town, South Ethiopia. Ethiop J Health Sci.

[37] Oyekale AS (2012). Factors influencing households’ willingness to pay for National Health Insurance Scheme (NHIS) in Osun state, Nigeria. Stud Ethno Med.

[38] Lasebew Y, Mamuye Y, Abdelmenan S (2017). Willingness to Pay for the Newly Proposed Social Health Insurance among Health Workers at St. Paul’s Hospital Millennium Medical College, Addis Ababa, Ethiopia. Int J Health Econ Policy.

[39] Gidey MT, Gebretekle GB, Hogan ME, Fenta TG (2019). Willingness to pay for social health insurance and its determinants among public servants in Mekelle City, Northern Ethiopia: a mixed methods study. Cost Eff Resour Alloc.

[40] Yeshiwas S, Kiflie M, Zeleke AA, Kebede M (2018). Civil servants' demand for social health insurance in Northwest Ethiopia. Arch Public Health.

[41] Mo H (2002). Health Insurance in Ethiopai Amharic version.

[42] Tewele A, Yitayal M, Kebede A. Acceptance for social health insurance among health professionals in government hospitals, Mekelle City, North Ethiopia. Adv Public Health. 2020;2020:6458425. 10.1155/2020/6458425.

[43] AZuogu BNM (2016). Level of awareness, and factors associated with willingness to participate in the National Health Insurance Scheme among traders in Abakaliki main market, Ebonyi State, Nigeria.

[44] Salameh AMM, Juni MH, Hayati K (2015). Willingness to pay for social health insurance among academic staff of a public University in Malaysia. Int J Public Health Clin Sci.

[45] Jofre-Bonet M, Kamara J (2018). Willingness to pay for health insurance in the informal sector of Sierra Leone. PLoS One.

